# Anti-TNF-Alpha Therapy and Systemic Vasculitis

**DOI:** 10.1155/2014/493593

**Published:** 2014-02-27

**Authors:** Pierre-André Jarrot, Gilles Kaplanski

**Affiliations:** ^1^Service de Médecine Interne, Hôpital de la Conception, 147 boulevard Baille, 13005 Marseille, France; ^2^INSERM UMR S 1076, UFR Pharmacie, Aix-Marseille-Université, 27 boulevard Jean Moulin, CS 30064, 13385 Marseille, Cedex 5, France

## Abstract

TNF-**α** is a pleiotropic cytokine, which plays a major role in the pathogenesis of numerous autoimmune and/or inflammatory systemic diseases. Systemic vasculitis constitutes a group of rare diseases, characterized by inflammation of the arterial or venous vessel wall, causing stenosis and thrombosis. Treatment of the different type of vasculitis mainly relies on steroids and immunosuppressive drugs. In case of refractory or relapsing diseases, however, a second line of treatment may be required. Anti-TNF-**α** drugs have been used in this setting during the last 15 years with inconsistent results. We reviewed herein the use of anti-TNF-**α** therapy in different kind of vasculitis and concluded that, except for Behcet's disease, this therapeutic option has not demonstrated significant improvement in the treatment of vasculitis.

## 1. Introduction

Tumour necrosis factor alpha (TNF-*α*) is a pleiotropic cytokine known to play a major role in host defense mechanisms, initiating a beneficial local inflammation which in excess, however, may cause tissue damage [1]. Since 1999, anti-TNF-*α* therapy has been used with success in the treatment of patients suffering from rheumatoid arthritis (RA), inflammatory enterocolitis (Crohn's disease and ulcerative colitis), spondyloarthropathies, or psoriasis [2–6]. Randomized international studies have shown the efficacy of five currently commercially available anti-TNF-*α* molecules. These molecules have also been tested in other autoimmune and inflammatory systemic diseases such as severe vasculitis refractory to conventional treatment but, to date, vasculitis are not included in the list of therapeutic indications of anti-TNF-*α* agents.

Systemic vasculitis is a group of rare diseases characterized by inflammation of the arterial or venous vessel wall, causing stenosis or thrombosis [7]. Initially classified by the size of the vessel involved, primitive vasculitis has been recently reclassified with the introduction of immunological markers in the new Chapel Hill Consensus classification [8]. One can distinguish between large vessels vasculitis (giant cell arteritis (GCA) and Takayasu arteritis (TA)), medium vessels vasculitis (periarteritis nodosa (PAN)), and small vessels vasculitis with immune complex deposits (mixed cryoglobulinemia (MC)) or associated with anti-neutrophil cytoplasmic antibodies (ANCA) (granulomatosis with polyangeitis (GPA) formerly Wegener granulomatosis, eosinophilic granulomatosis with polyangeitis (EGPA) formerly Churg-Strauss disease, and micropolyangeitis (MPA)). In addition, some diseases may affect vessels of variable size (Behcet disease (BD)) [8]. We reviewed the published experience related to the use of anti-TNF-*α* therapy in these diseases, pointing to the fact that data are relatively rare and often contradictory.

## 2. Rationale for the Use of Anti-TNF-**α** in Vasculitis?

Two forms of TNF-*α* are synthesized by activated macrophages and dendritic cells: a transmembrane precursor form (26 Kda) which is proteolytically cleaved in a soluble form (17 kda) by a TNF-*α* converting enzyme (TACE) [9]. These two forms bind to two ubiquitous cell surface receptors (TNFR1 and TNFR2) on target cells to initiate proinflammatory genes transcription via the activation of Nuclear Factor Kappa B (NF*κ*B) and Mitogen Activated Protein (MAP) Kinase pathways, as well as proapoptotic genes transcription by the induction of death signal pathways [1, 10]. TNF-*α* induces leukoendothelial adhesion via increased expression of various adhesion molecules, such as E-selectin, Intercellular Adhesion Molecule 1 (ICAM-1), and Vascular Adhesion Molecule 1 (VCAM-1), and mediates tissue leukocyte infiltration through chemokine synthesis [11]. TNF-*α* induces metalloproteinase production and may also participate in endothelial cell death directly via apoptosis or indirectly via neutrophil activation [10].

In addition, TNF-*α* may play a role in neutrophil “priming” inducing membrane expression of proteinase-3 or myeloperoxidase, which are subsequently recognized by ANCA in ANCA-associated vasculitis (AAV) [12]. This cytokine may thus be involved in the pathogenesis of different kind of vasculitis. In addition, binding of anti-TNF-*α* to membrane-associated TNF-*α* can have an agonistic action, initiating reverse signaling and processes such as apoptosis, cytokine suppression, and cell activation, which could constitute an interesting target in the treatment of vasculitis [11, 13]. To date, 5 different anti-TNF-*α* drugs have been developed and are commercially available, 3 consisting in monoclonal antibodies (infliximab (IFX), adalimumab (ADA), and golimumab). IFX is usually used intravenously at 3 to 5 mg/kg every 8 weeks, and ADA and golimumab are used subcutaneously, 40 mg every 2 weeks for the former and 50 mg once a month for the latter. The fourth available drug is a dimer of a chimeric protein genetically engineered by fusing the extracellular ligand binding domain of human tumour necrosis factor receptor 2 (TNFR2/p75) to the Fc domain of human IgG-1 (etanercept (ETN)) and is used subcutaneously at 25 mg twice a week. The last is a humanised Fab fragment conjugated to polyethylene glycol (certolizumab pegol) but has never been used in vasculitis. Monoclonal antibodies and certolizumab are active on the two molecular forms of TNF*α*, whereas etanercept neutralizes soluble TNF-*α* only.

## 3. Large Vessels Vasculitis

### 3.1. Giant Cell Arteritis (GCA)

The pathogenesis of GCA seems due to an abnormal cell-mediated immune response taking place in the vessel wall, leading to macrophage activation, giant cell formation, and excess production of interferon gamma [14]. Other proinflammatory cytokines such as IL-1, IL-6, and IL-17 may be involved in GCA pathogenesis, whereas experimental data showing the role of TNF-*α* in this disease are sparse [15]. GCA mostly affects people older than 50 years. Long-term corticosteroids remain the main treatment which is unfortunately commonly complicated by many side effects [16]. Immunosuppressive drugs such as methotrexate (MTX) or aziatropine (AZT) have been used in order to have a steroid sparing effect and in some corticodependent/resistant patients. MTX was tested in 3 prospective studies with contradictory effects, and AZT gave disappointing results in a controlled study enrolling 31 patients [17–20]. Thus, after a few cases showing successful anti-TNF-*α* treatment in corticodependent GCA patients have been reported, a comparative double blind study was conducted using IFX but was subsequently stopped due to the lack of efficacy on the prevention of relapse [21]. Regarding ETN, a randomized controlled study against placebo was conducted on 17 patients and demonstrated a significant corticosteroid sparing effect after one year, but not for a longer period which was nevertheless the primary end point [22]. Finally, ADA showed no benefit in a prospective study including 70 patients with a primary end point of steroid sparing at week 26 [23]. In view of these different studies, anti-TNF-*α* therapy is not recommended in GCA (Table 1).

### 3.2. Takayasu Arteritis (TA)

TA is characterized by inflammation of large vessels, primarily the aorta and its main branches, resulting in anevrysm formation, vascular stenosis, or occlusion, affecting mainly young women. TA is a chronic idiopathic granulomatous panarteritis, resulting from infiltration of the three layers of the vessel wall by macrophages, T lymphocytes, and natural killer cells [14, 24]. First line treatment consists in high doses of corticosteroids [24]. However, almost 50% of TA patients demonstrate glucocorticoid resistance or relapsing disease, requiring the addition of immunosuppressive agents such as AZT, cyclophosphamide (CYC), or mycophenolate mofetil (MMF) with inconsistent efficiency [25]. To date, approximately 120 TA patients have been treated with anti-TNF-*α*, mostly with IFX, as a second/third line immunosuppressive therapy. A first open label prospective study involving 15 patients with active or relapsing disease (8 treated by infliximab and 7 by etanercept) suggested some therapeutic efficacy which was confirmed by other studies (90% rate of remission and 60% for sustained remission) [26–28] (Table 2). Relapses, however, seem to be common after the drug is stopped following remission [27]. In summary, despite the fact that no prospective controlled study has been conducted to date, anti-TNF-*α* may constitute an interesting therapeutic option in TA patients who have been unable to achieve or maintain remission with steroids alone or common immunosuppressive agents.

## 4. Medium Size Vessels Vasculitis

### 4.1. Periarteritis Nodosa (PAN)

PAN is a rare necrotizing vasculitis complicating hepatitis B virus chronic carriage affecting medium size vessels, whose incidence has declined since the introduction of hepatitis B vaccination and antiviral treatments [29]. The treatment of PAN consists in steroids or immunosuppressive drugs in association with antiviral therapy, according to the gravity of the disease [29, 30]. In a few case reports, IFX has been used in refractory forms of the disease or because of intolerance of conventional drugs and seems to be effective [31].

## 5. Small Size Vessels Vasculitis

### 5.1. Mixed Cryoglobulinemia (MC)

MC is a small vessel vasculitis involving skin, joints, peripheral nerves, and the kidney, which is mainly associated with hepatitis C, Sjogren's syndrome, or lymphoma [32]. MC is a model of immune-complex-mediated inflammation of blood vessels and may involve TNF-*α* [33]. Before the area of anti-CD-20-targeted biotherapy, only 3 patients with refractory hepatitis C-associated MC treated with anti-TNF-*α* have been reported with conflicting results, some patients even experiencing severe flare-up of the disease [34, 35]. At present, the treatment of MC relies on plasma exchange, immunosuppressive drugs, antivirals, and anti-CD-20 therapy (rituximab) [36].

### 5.2. ANCA-Associated Vasculitis (AAV)

AAV is a group of multisystemic diseases characterized by a pauci-immune small vessel vasculitis which includes three different entities: two recently renamed granulomatosis with polyangeitis (GPA), eosinophilic GPA (EGPA), and microscopic polyangeitis (MPA). AAV pathogenesis is consistent with a primary role for neutrophils, which are both the effector cells responsible for endothelial damage via oxygen radical synthesis and enzyme degranulation and the targets of ANCA [37]. Current AAV treatment is based on a six-month induction phase associating high-dose steroids with immunosuppressive drugs such as CYC or rituximab, followed by an 18-month maintenance therapy with AZT [38]. However, using these standard treatment protocols, relapses are very common, occurring in 49% and 35% of patients with GPA and MPA, respectively [29, 39]. Moreover, some patients remain refractory to conventional treatments, raising the need for new therapeutic options.

Etanercept was tested initially in an open label trial including 20 relapsing or incompletely controlled GPA and seemed efficient with a 3-point reduction of the Birmingham Vasculitis activating (BVAS) score at 6 months [40]. These results were not confirmed in a larger controlled prospective study including 180 GPA patients [41]. However, it should be noted that this latter study had some design limitations since the two groups were not homogeneous at the baseline, and some patients had localized forms of the disease. Finally, although ETN is known to be of little usefulness in granulomatous disease, only the WGET study provides data confirming that the addition of ETN to usual treatments is ineffective in the maintenance regimen of GPA [41]. Anti-TNF-*α* monoclonal antibodies have also been tested in refractory AAV patients. The efficiency of infliximab has been observed in prospective observational studies [42, 43]. One multicentric prospective randomized control trial involving 17 patients compared the efficacy of infliximab (*n* = 9) or rituximab (*n* = 8) in association with steroids and immunosuppressive drugs in refractory GPA for a median follow-up of 30.6 months (+/−15.4). Efficacy of infliximab and/or rituximab to obtain remission at one year was observed, with an advantage for rituximab. During long-term follow-up, rituximab was also more effective at obtaining and maintaining remission [44]. Recently, 33 patients with active AAV (BVAS > 10) were enrolled in an open prospective trial to study infliximab adjunction to standard therapy in order to obtain remission for a median follow-up of 12 months and demonstrated no benefit with anti-TNF-*α*. However, this was a noncontrolled study and groups lacked homogeneity [45]. The last open label prospective study was conducted with adalimumab associated with standard therapy in refractory AAV with renal involvement. Although no significant gain in response rate was observed, a significant steroid sparing effect was noted [46].

In conclusion, it seems that a short course anti-TNF-*α* therapy using infliximab may be useful in complement to standard regimen in some refractory AAV patients, but the recent published data on the beneficial effects of rituximab for obtaining remission and preventing relapses in MPA and GPA appear much more convincing and may significantly limit the use of anti-TNF-*α* treatment in AAV in the future [47, 48] (Table 3).

## 6. Variable Size Vessels Vasculitis

### 6.1. Behcet's Disease (BD)

BD is a chronic and relapsing systemic inflammatory disorder characterized by recurrent orogenital ulcerations with possible mucocutaneous, ocular, digestive, vascular, and/or central nervous system involvements. BD pathogenesis is still unclear, but an association between genetic intrinsic factors (HLA-B5) and triggering extrinsic factors is suspected [49, 50]. First line treatments are adapted to each organ involvement, such as colchicine for mucocutaneous symptoms or combination of steroids and immunosuppressive drugs in case of severe visceral involvement [51]. Some patients however may develop severe and even life-threatening complications despite these standard treatments regimens. The pathogenic role of TNF-*α* in mediating tissue injury during BD seems to be major, and increased levels of TNF-*α* and soluble TNF receptors have been found in the peripheral blood of patients with active BD [52, 53].

In 2001, anti-TNF-*α* treatments were first tested for severe eye involvement showing promising results [54]. A meta-analysis collecting 158 patients included in 14 prospective studies testing infliximab in BD with ocular lesions refractory to immunosuppressive drugs was realized in 2011. A complete or partial remission was achieved in 65% and 24% of patients receiving infliximab, allowing glucocorticoids and immunosuppressive release in about 40% [55]. A recent open label prospective study enrolling 63 patients receiving infliximab showed similar results after one year of treatment [56]. Interestingly, the improvement was rapidly obtained following the initiation of infliximab [57].

Five open prospective studies demonstrated a beneficial effect of long-term infliximab treatment on the prevention of relapse, maintenance of visual acuity, and immunosuppressive drugs tapering [57–61] (Table 4). Intravitreous injection of infliximab was also tested in refractory uveitis in 15 patients and demonstrated a positive effect [62]. Regarding the use of adalimumab, etanercept, and golimumab, only case reports are currently available but showed beneficial effects in refractory uveitis, [63–65].

Anti-TNF-*α* treatments were tested in severe refractory cutaneous manifestations, especially etanercept which revealed a significant efficacy compared to placebo on oral ulcers, and nodular lesions [66].

Infliximab in monotherapy was also tested in refractory entero-Behcet during a prospective open trial and showed 100% improvement on clinical, CT-scan, and colonoscopy [67]. Adalimumab may also be efficient in this kind of patients [68].

In addition, infliximab has been used in BD affecting the central nervous system (CNS) in open prospective studies and was almost always successful [69, 70]. These patients were refractory to high-dose steroids combined with various immunosuppressive drugs and demonstrated major improvement or stabilization of their symptoms. Long-term remission (6–18 months) after discontinuation of infliximab therapy was noted in 75% of patients [69, 70] (Table 5). Adalimumab and etanercept have also been tried in case reports of BD with CNS involvement with a favorable outcome [71, 72]. In case of a first anti-TNF-*α* failure in refractory BD, a switch of molecule was made in up to 25% of cases with a 70% improvement [73].

In summary, infliximab seems effective in induction treatment and relapse prevention in severe BD refractory to glucocorticoids or immunosuppressive drugs, especially in case of eye involvement. It could also be an alternative therapy to immunosuppressive drugs in case of central nervous system or gastrointestinal manifestations. Nevertheless, the main limitation of the present analysis is that most information originated from limited cases or noncontrolled studies, strongly raising the need for properly randomized controlled clinical trials.

## 7. Safety and Tolerance of TNF-Alpha Blockade

In vasculitis, anti-TNF-*α* are often prescribed as second/third line treatments in patients already immunocompromised by long-term use of glucocorticoids and immunosuppressive drugs. Despite this fact, side effects have been reported up to 46% and are mostly moderate. The literature review suggests that these drugs are rather safe, in agreement with what is known in RA and spondyloarthropathy [56, 74].

Patients treated with anti-TNF-*α* are prone to develop soon after initiation bacterial and viral infections mostly affecting respiratory or urinary tracts and cutaneous or soft tissues. Furthermore, reactivation of latent tuberculosis or extrapulmonary forms of this infection is another well-known threat [75–81].

The role of anti-TNF-*α* therapy in carcinogenesis and tumor progression remains a matter of controversy. A large study assessed the risk of cancer in a RA cohort treated with anti-TNF-*α* and showed a relative risk (RR) of 1.00 (95% CI: 0.86–1.15) compared to the biotherapy naïve RA cohort. RR did not increase with longer exposure or with the cumulative duration of active anti-TNF-*α* therapy during a 6-year follow-up period [82]. Regarding the risk of hemopathy, infliximab and etanercept were not associated with the occurrence of lymphoma in a study involving 19,000 patients with RA [83]. One observational study, however, showed a positive association between anti-TNF-*α* therapy in RA and nonmelanoma skin cancers with a follow-up period of 3 years [84]. In vasculitis, especially AAV, an unusually high frequency of solid cancer was reported in a randomized controlled trial that evaluated etanercept for maintenance of remission in 180 patients with GPA. But all the patients (*n* = 6) who developed cancer also received standard therapy associating MTX or CYC, which are known to be involved in cancerogenesis [44]. Without large specific epidemiological studies, caution is however advised.

Besides infection and malignancy, anti-TNF-*α* treatments can induce acute infusion reaction, which may lead to discontinuation of the treatment. Anti-TNF therapy may also favor antinuclear antibodies appearance which are, however, weakly associated with clinical symptoms [85, 86]. Other extending complications are allocated to anti-TNF-*α* treatments, such as the occurrence of demyelinating and sarcoid-like granulomatous diseases [87].

## 8. Conclusion

Anti-TNF-*α* treatments in vasculitis did not demonstrate the same efficacy as in other inflammatory diseases such as RA. Nevertheless, anti-TNF-*α* should be used in the treatment of BD refractory to immunosuppressive drugs, especially in case of ocular, CNS, or digestive tract involvement. Some interesting data are also available for the use of anti-TNF-*α* treatments in refractory AAV, but the recent reports on rituximab efficacy in these diseases and the relative innocuity of this drug may limit the use of anti-TNF-*α* in these diseases, in the future. In both BD and AAV, an anti-TNF monoclonal antibody especially infliximab should be preferred to etanercept. In addition, physicians must be aware of the risk of infection using these drugs, especially in patients already immunocompromised by previous treatments. Regarding other vasculitis, published data are not in favor of efficiency of anti-TNF-*α* which, therefore, should not be used.

## Figures and Tables

**Table 1:** Randomized controlled trials performed in GCA.

References	Design/anti-TNF-*α* therapy	Number of patients	Main objectives	Follow-up	Main results	Side effects
Hoffman et al. [21]	Randomized controlled trial IFX versus placebo	44 (28 IFX, 16 placebo)	Free of relapses and adverse events at 54 weeks	54 weeks	Stopped early at week 22 for lack of efficacy (43% for IFX versus 50% for placebo)	Infection: 71% for IFX versus 56% for placebo (NS)

Martínez-Taboada et al. [22]	Randomized controlled trial ETN versus placebo	17 (8 ETN, 9 placebo)	To withdraw the corticosteroid therapy at 12 months	12 months	50% for ETN versus 22% for placebo (NS)	No differences between the two groups

Mariette et al. [23]	Randomized controlled trial ADA versus placebo	70 (34 ADA, 36 placebo)	Remission and corticosteroid < 0,1 mg/kg/day at 26 weeks	26 weeks	55,9% for ADA 50% for placebo (NS)	Severe infections: 8,8% for ADA versus 13,9% for placebo (NS)

ns: nonsignificant.

**Table 2:** Open label trial performed in TA.

References	Design/anti-TNF-*α* therapy	Number of patients	Main objectives	Follow-up	Main results	Side effects
Hoffman et al. [26]	Open label trial IFX/ETN	15	Remission and discontinuation of corticosteroids	12 months	67% complete remission 27% partial remission (50% of glucocorticoid requirements reduction)	One infusion reaction to IFX

**Table 3:** Open label and randomized controlled trials performed in AAV.

References	Design/anti-TNF-*α* therapy	Number of patients	Main objectives	Follow-up	Main results	Side effects
Stone et al. [40]	Open label trial ETN	20 GPA	BVAS at 6 months adverse events during 6 months	6 months	3 points Decrease of BVAS (*P* < 0,05)	Injection site reaction in 25% 95% of patients still taking ETN

WGET research group [41]	Randomized controlled trial ETN versus placebo	180 GPA (89 ETN, 91 placebo)	Sustained remission at 27 months (BVAS = 0)	27 months	69,7% for ETN versus 75,3% for placebo (NS)	56,2% for ETN versus 57,1% for placebo had a life threatening event (NS)

Morgan et al. [45]	Open label trial IFX	33 (22 GPA, 11 MPA) (16 IFX, 17 standard treatment)	Time to clinical remission (BVAS ≤ 1)	12 months	No difference between the two groups	Infections in 8 patients for IFX and 7 for standard treatment (NS)

De Menthon et al. [44]	Randomized controlled trial IFX versus rituximab	17 GPA (9 IFX, 8 RTX)	CR/PR at month 12	12 months	IFX: 2 CR, 1 PR RTX: 4 CR, 1 PR	One death in both groups (invasive *Aspergillosis* for IFX and sudden death for RTX)

Laurino et al. [46]	Phase 2 open label trial ADA	14 (9 GPA, 5 MPA)	(i) Induction of remission within the first 14 weeks (ii) time to remission	17 months	(i) 78,5% achieved remission (ii) Time to remission 12 weeks	Infections in 3 patients (1 mild and 2 severe including 1 death)

NS: Nonsignificant; RTX: rituximab; CP: complete remission; PR: partial remission.

**Table 4:** Open label and randomized controlled trials performed in Behcet's uveitis.

References	Design/anti-TNF-*α* therapy	Number of patients	Main objectives	Follow-up	Main results	Side effects
Okada et al. [56]	Open label trial IFX	63	Efficacy of IFX in the first year of treatment	12 months	Improvement in 69% Improvement somewhat in 23% Unchanged in 8%	46% of side effects including 3 infusion reactions No serious side effects

Sfikakis et al. [57]	Open label trial IFX	25	Remission at day 28	28 days	89% of complete remission	No serious side effects

Ohno et al. [58]	Open label trial IFX	12	Frequency of ocular attacks	14 weeks	Reduction in the number of relapses for IFX (5 mg/kg and 10 mg/kg)	One case of tuberculosis (IFX 10 mg/kg)

Accorinti et al. [59]	Open label trial IFX	12	Frequency of ocular attacks	15 months	91% of reduction in the number of relapses	33% of side effects including one tuberculosis and one herpetic keratitis

Tognon et al. [60]	Open label trial IFX	7	Frequency of ocular attacks	23 months	21 to 6 ocular attacks observed in the equivalent period of time before treatment	One infusion reaction

Tugal-Tutkun et al. [61]	Open label trial IFX	13	Absence of ocular attacks	6 years	31% remained attack-free 32 to 13 ocular attacks observed in the equivalent period of time before treatment	No serious side effects (7 respiratory tract infection and one infusion reaction)

**Table 5:** Open label and randomized controlled trials performed in BD with cutaneous, intestinal, and central nervous system involvements.

References	Type of involvement/design/anti-TNF-*α* therapy	Number of patients	Main objectives	Follow-up	Main results	Side effects
Melikoglu et al. [66]	Cutaneous/randomized controlled trial/ETN versus placebo	40 (20 ETN/20 placebo)	(i) Pathergy response and monourate sodium status (ii) frequencies of mucocutaneous manifestations	4 weeks	(i) No differences between the two groups (ii) decrease of frequency of mucocutaneous manifestations	No serious side effects

Iwata et al. [67]	Entero-Behcet/open label trial/IFX	10	Clinical manifestations CT-scan	12 months	Rapid and dramatic improvement for all the patients	No serious side effects

Kikuchi et al. [69]	Neuro-Behcet/open label trial/IFX	5	Clinical manifestations brain magnetic resonance imaging	24 weeks	Improvement in 3 patients	One pneumocystis pneumonia

Giardina et al. [70]	Neuro-Behcet/open label trial/IFX	21	Clinical manifestations (CR/PR)	54 weeks	85% of CR 9% of PR	No serious side effects

CR: complete remission; PR: partial remission.
